# A panel of oxidative stress assays does not provide supplementary diagnostic information in Behcet's disease patients

**DOI:** 10.1186/1476-9255-9-13

**Published:** 2012-04-03

**Authors:** Yasemin D Akcay, Ferhan G Sagin, Kenan Aksu, Gokhan Keser, Emma Taylor, Iona Knight, Paul G Winyard, Eser Y Sozmen

**Affiliations:** 1 Department of Biochemistry, Ege University, School of Medicine, 35100 Bornova, Izmir, Turkey; 2 Department of Rheumatology, Ege University, 35100 Bornova, Izmir, Turkey; 3 Peninsula Medical School, University of Exeter, Exeter EX1 2 LU, UK

**Keywords:** Behçet's disease, inflammation, cytokines, S-nitrosothiols, nitrotyrosine, electron paramagnetic resonance spectrometry, nitric oxide, oxidative stress

## Abstract

**Background:**

Recent findings suggest a role of oxidative stress in the pathogenesis of Behcet's disease (BD), but the utility of oxidative stress-associated assays in offering diagnostic information or in the monitoring of disease activity is largely unassessed.

**Objective and methods:**

We aimed to measure oxidative and inflammatory markers, along with the markers of reactive nitrogen species, S-nitrosothiols and 3-nitrotyrosine, in BD patients (n = 100) and healthy volunteers (n = 50). These markers were evaluated in regard to their role in the pathogenesis of BD as well as their relation to clinical presentation, disease activity and duration.

**Results:**

Median values for erythrocyte sedimentation rate (ESR), C-reactive protein, leukocyte count, and IL-18 levels, as well as myeloperoxidase (MPO) activity, were statistically higher in the patient group compared to controls. Some inflammation markers (ESR, neutrophil and leukocyte counts) were statistically higher (p < 0.05) in the active period. In contrast, oxidative stress-associated measures (erythrocyte lipid peroxidation, antioxidant enzymes and measures of serum antioxidant capacity), revealed no statistically significant differences between the median values in BD patients versus healthy control subjects (p > 0.05 in all statistical comparisons), nor was there any difference in median levels of these oxidative stress markers in active disease versus disease remission. S-nitrosothiols and 3-nitrotyrosine were undetectable in BD plasma.

**Conclusions:**

The application of oxidative stress-associated measures to BD blood samples offered no supplemental diagnostic or disease activity information to that provided by standard laboratory measures of inflammation. S-nitrosothiols and 3-nitrotyrosine appeared not to be markers for active BD; thus the search for biochemical markers that will indicate the active period should be continued with larger studies.

## Background

Behcet's disease (BD) is a multisystem, chronic inflammatory, relapsing disorder that is characterized by oral and genital ulcerations and ocular, arthritic, vascular and neurological involvement. Its diagnosis is generally clinical with a higher endemic rate in Turkey, Iraq, Iran, Korea and Japan. There is no biochemical parameter showing the active phase of the disease, other than nonspecific tests of CRP, erythrocyte sedimentation rate and increased leukocyte count [1]. Although the etiology is unclear, viruses, autoimmune mechanisms and toxic agents have been suggested [1]. Recent findings indicated that oxidative stress and an insufficient antioxidant system might have roles in BD pathogenesis. It has been suggested that increased phagocytic cell activity in BD increases reactive oxygen and nitrogen species [2] which lead to severe inflammatory and degenerative pathological events by oxidative reactions with DNA, proteins, fatty acids and polysaccharides [3].

Vascular inflammation is one of the important features of BD [1,4]. NO is an important mediator of immunity and inflammation [2]. Thus NO is associated with various inflammatory and autoimmune diseases such as rheumatoid arthritis [5,6]. However, determination of the plasma levels of NO is difficult due to the instability of the molecule and its conversion to reaction products: nitrite (NO_2_^-^), nitrate (NO_3_^-^), S-nitrosothiols (RSNOs) and 3-nitrotyrosine (3-NT). Recently increases in nitrite and nitrate concentrations have been shown in BD patients and were associated with disease activity [7,8]. However, there is limited information on the potential role of RSNO and 3-NT formation in BD.

In the present study we assessed, in BD patients, standard measures of inflammation (CRP, ESR, leukocyte count), proinflammatory cytokine levels (TNF-α, IL-18), Trolox equivalent antioxidant capacity (TEAC), ferric reducing antioxidant power (FRAP), total antioxidant enzyme activity (AOA), catalase (CAT), superoxide dismutase (SOD) and myeloperoxidase (MPO) activities. RSNO and 3-NT levels were also investigated. All these markers were evaluated in regard to their relation to the disease activity and clinical presentation in BD patients, as well as their role in the pathogenesis of BD. It was anticipated that the results could help to identify a parameter that can be helpful in clinical practice to determine disease activity.

## Methods

### Subjects

One hundred patients with BD (36 female, 64 male; age mean ± S.D. 39.1 ± 9.7 years), and 50 sex-matched (20 female, 30 male) and age-matched (38.0 ± 6.6 years) healthy volunteers as control subjects were included in the study. All BD patients fulfilled the diagnostic criteria of the International Study Group for BD [9]. This means that every single patient had oral ulcers plus two of the other four criteria. However, 13 patients fulfilled these criteria only with mucocutaneous findings, i.e. oral and genital ulcers plus relevant skin lesions. On the other hand, 27 patients had uveitis as the dominant clinical feature. Among the other clinical features not included in the diagnostic criteria, vascular involvement and articular involvement were additionally present in 21 and 18 BD patients, respectively. The remaining 18 patients had combinations of various involvements of BD. Patients were also divided into 2 groups, as the active and remission groups. At the time of the clinical assessment, patients were included in the active group if they had at least two of the following clinical findings: mouth ulcers, genital ulceration, active uveitis, skin findings, recent arthritis and vascular involvement. Among the 100 patients, 74 were in the remission period, and the others (n = 26) were in an active period. for BD [9]. The study was approved by the Ethics Committee of Ege University Medical School and informed consent was obtained from all subjects.

### Blood sample preparation

Blood samples were collected in heparin-containing tubes, EDTA-containing tubes and anticoagulant-free tubes after an overnight fast. Plasma and serum were separated immediately. Erythrocyte lysates were prepared [10] and polymorphonuclear (PMN) leukocytes were isolated [11] from the heparin-containing tubes. For S-nitrosothiol analysis, 0.7 mL plasma was added to an aqueous solution of 250 μM N-ethylmaleimide (NEM; 10 μL: Sigma-Aldrich, E1271-5 G) for 20 min before the storage of the samples [5]. Samples were stored at -80° until analysis.

### Serum analyses

TNF-α and IL-18 levels were measured using enzyme-linked immunosorbent assay (ELISA) kits (Biosource, Cat. No.: KHC3011, USA, Biosource, Cat. No.: KHC0181, USA) according to the manufacturers' instructions. AOA (total antioxidant activity) was determined spectrophotometrically [12]. A solution of 0.1 mM 1,1-diphenyl-2-picrylhydrazil was rapidly mixed with the sample (1/10, v/v). The decline in absorbance was recorded at 550 nm against an ethanol blank over a period of 15 min in a microplate reader (Thermo Labsystems, Multiskan EX instrument, which was also used for all subsequent spectrophotometric assays). The decrease in absorbance corresponding to 100% radical scavenging was determined with a solution of pyrogallol in dimethyl sulfoxide (ca. 0.5%), which caused complete scavenging within seconds.

TEAC was determined spectrophotometrically [13]. ABTS 2,2'-azinobis3-ethylbenzthiazolinesulfonate; 7 mM) and potassium persulphate (4.95 mM) were mixed (1/1 v/v) and stored at room temperature for 12 h before use. This reactant was diluted with phosphate buffer (1/25 v/v) until the absorbance value reached up 1.0-1.5. The working solution (975 μL) was mixed with 5-25 μL serum and absorbances were read at 734 nm wavelength. Phosphate buffer and Trolox were used for controls and standards, respectively.

FRAP was determined spectrophotometrically [14]. A mixing solution (10:1:1, v/v/v) of acetate buffer (10 mM, pH = 3,6), 2,4,6-tripyridyl-S-triazine (TPTZ; 10 mM) and FeCl_3 _(20 mM) was added to the serum sample and incubated at room temperature for 30 min. Readings were done at 620 nm.

### Plasma analyses

RSNO concentrations were measured using a previously described EPR spectrometry method [5] in 15 plasma samples. In this technique the RSNOs were degraded at an alkaline pH (pH 10,4), and the NO^• ^released was measured in the presence of the spin trap complex (MGD)_2_- Fe^2+^, in which MGD is *N*-methyl-D-glucamine dithiocarbamate. In order to concentrate S-nitrosothiols and further improve the detection limit for S-nitrosothiols, the EPR method was modified using different techniques such as ultrafiltration (Whatman ultrafiltration devices), incubation of the samples at different temperatures (room temperature, 50°C) and different time intervals (1, 2, and 5 min) and enzymatic proteolysis was carried out using proteinase K and pronase. Each procedure was performed several times.

Nitrotyrosine (3-NT) concentrations were investigated with an enzyme-linked immunosorbent assay using a commercial kit (Hycult Biotechnology, The Netherlands; Cat. No.: HK501). This 3-NT ELISA test kit is based on the sandwich method. All samples were diluted 1:20 prior to conducting the assay.

### Blood cell-based assays

#### Isolation of polymorphonuclear (PMN) leukocytes and MPO assay

Heparinized blood samples were diluted with an equal volume of phosphate buffered saline (PBS) and then 4 mL of Ficoll Hypaque (Sigma-Aldrich-1077) was added to each sample and centrifuged for 20 min at 1200 g. After centrifugation, the leukocyte pellet was taken and mixed with 0.9% NaCl. This mixture was centrifuged for 5 min at 1500 g. The cell pellet that contained the PMN was then resuspended in phosphate buffer and stored at -80°C until analysis. Leukocyte MPO activities were measured according to the modified method of Grisham et al. [15]. Briefly, following homogenization, leukocyte pellets were rehomogenized in 0.5% HETAB (hexadecyltrimethyl ammonium bromide) in phosphate buffer (50 mM, pH = 6.0). Following three freeze-thaw cycles, samples were centrifuged at 10,000 rpm for 10 min. Supernatants were added to a reaction solution containing 0.5 M *o*-dianisidine (in phosphate buffer). After addition of hydrogen peroxide solution (20 mM), absorbances of samples were recorded at 492 nm with a microplate reader during 3 minutes with 15 s intervals. Protein concentration was measured according to Lowry's method [16].

#### Preparation of erythrocyte haemolysates, followed by eSOD, eCAT and eTBARS assays

After separation of plasma, the packed erythrocytes were washed twice with 9 g⁄L NaCl and haemolysed with ice-cold water (1/5, v/v) Erythrocyte SOD (eSOD), eythrocyte catalase (eCAT) and erythrocyte TBARS (e-TBARS) activities were determined immediately in haemolysates. The haemoglobin values were measured by Drabkin's method [17].

Erythrocyte SOD (eSOD) activity was measured spectrophotometrically using a commercial kit (RANDOX, UK, cat no. SD 125). This method is based on the use of xanthine and xanthine oxidase (XOD) to generate superoxide radicals which react with a chromogen solution to form a red formazan dye. SOD activity is then measured by the degree of inhibition of this reaction by means of the decrease in absorbance at 490 nm. This result was normalised to the Hb concentration, and SOD activity was expressed as units/g Hb.

Erythrocyte CAT (eCAT) activities were determined as described by Sozmen et al. [7], in which the degradation of H_2_O_2 _was recorded spectrophotometrically at 240 nm. One unit of CAT was defined as the amount of enzyme that decomposes 1 μm0l H_2_O_2 _/min.

Polyunsaturated fatty acids are oxidized due to increased oxidant stress resulting in high plasma TBARS levels. Erythrocyte TBARS (eTBARS) levels were determined according to the method previously reported [18]. After dilution of hemolysates with physiologic saline, they were incubated with TBA-working solution (0.12 M TBA in 15% TCA and 1% HCl) for 30 min at 95°C. eTBARS concentrations were calculated using a calibration curve constructed from 1,1,3,3- tetraethoxypropane.

### Statistical analysis

All statistical analyses were performed using GraphPad Prism v5.04. Data were not normally distributed, and were expressed as median values with interquartile ranges. Data were analyzed by the Kruskal-Wallis test, and Mann-Whitney U test to compare the median values of two groups. Correlations between variables were tested using Spearman's rank correlation. P values less than or equal to 0.05 were evaluated as statistically significant.

## Results

The results of all parameters, when comparing the group of healthy subjects (n = 50) with all BD patients analysed (n = 100) are given in Table 1 except the plasma RSNO (n = 15) and 3-NT (n = 53) concentrations which were below the detection range in both patient and healthy control groups. As expected, measures of inflammatory response, i.e. ESR, CRP, leukocytes, IL-18 and MPO activity, were statistically higher in the patient group compared with control group (all p < 0.0001). In contrast, oxidative stress-associated measures, i.e. e-TBARS, e-SOD, e-CAT, AOA, FRAP and TEAC, revealed no statistically significant differences between the median values in patients versus healthy control subjects (p > 0.05 in all statistical comparisons).

**Table 1:** Standard laboratory indices and oxidative stress-associated measures in BD patient and healthy control groups.

	Control Subjects		BD Patients		P value
ESR (1 h)	7.0 (5.0-10.0)	n = 50	11.5 (7.0-21.3)	n = 100	**< 0.0001**

CRP (mg/L)	0.09 (0.05-0.11)	n = 50	0.36 (0.10-2.20)	n = 98	**< 0.0001**

Leukocytes(10^3^/mm^3^)	5300 (4800-6200)	n = 43	7250 (6100-9300)	n = 98	**< 0.0001**

TNF-α (pg/mL)	2.74 (1.26-3.70)	n = 33	3.43 (1.76-4.70)	n = 82	0.093

IL-18 (pg/mL)	181.4 (117.2-245.6)	n = 37	263.4 (193.9-375.3)	n = 100	**< 0.0001**

MPO (U/mg protein)	1.19 (0.45-2.99)	n = 37	3.86 (1.70-5.86)	n = 93	**< 0.0001**

e-TBARS (nmol/gHb)	27.1 (23.7-29.6)	n = 38	28.4 (25.0-32.9)	n = 100	0.175

e-SOD (U/gHb)	674.4 (417.4-913.4)	n = 38	621.4 (427.5-1026.0)	n = 99	0.838

e-CAT (U/gHb)	4711 (3968-5373)	n = 38	5105 (4419-5914)	n = 100	0.110

AOA (%)	34.9 (27.3-49.5)	n = 40	42.1 (31.8-51.3)	n = 89	0.304

FRAP (μmol/L)	864.9 (728.5-1050.0)	n = 33	773.0 (675.3-896.6)	n = 99	0.056

TEAC (trolox equivalent)	4.69 (4.01-4.98)	n = 33	4.68 (4.20-5.18)	n = 100	0.560

Data are expressed as median (interquartile range) values

ESR = Erythrocyte sedimentation rate, CRP = C-reactive protein, TNF-α = tumor necrosis factor-α, IL = interleukin, MPO = myeloperoxidase, e-TBARS = erythrocyte thiobarbituric acid reactive substances, e-SOD = erythrocyte superoxide dismutase, e-CAT = erythrocyte catalase, AOA = antioxidant activity, FRAP = ferric reducing antioxidant power, TEAC = Trolox equivalent antioxidant capacity

Results of all parameters analysed in regard to active (n = 26) and remission (n = 74) phases of the disease are given in Table 2. The median values for ESR, CRP and leukocyte count were statistically higher in active disease patients, compared with patients in remission (p < 0.0001, p < 0.0001 and p = 0.004, respectively), while none of the other parameters showed statistically significant increases in the active disease group compared with the patients in remission (p > 0.05). Indeed, the median serum TNF-α concentration showed a trend (p = 0.06) towards being lower in patients with active disease than those in remission.

**Table 2:** Results in regard to active and remission phases of the disease.

	Remission		Active		P value
Age	41.22 ± 9.50	n = 74	32.96 ± 7.80	n = 26	**0.0001**

Disease duration (years)	9.5 (5.0-15.0)	n = 74	6.5 (4.0-12.0)	n = 26	**0.046**

ESR (1 h)	9.0 (6.0-17.0)	n = 73	27.0 (11.5-49.5)	n = 25	**< 0.0001**

CRP (mg/L)	0.22 (0.10-0.80)	n = 73	2.70 (1.02-6.20)	n = 25	**< 0.0001**

Leukocyte (10^3^/mm^3^)	7100 (5850-8450)	n = 73	9000 (7200-11800)	n = 25	**0.004**

TNF-α (pg/mL)	3.63 (1.99-5.26)	n = 66	1.99 (1.34-3.90)	n = 16	0.060

IL-18 (pg/mL)	280.0 (202.0-379.0)	n = 74	213.4 (171.5-306.7)	n = 26	**0.040**

MPO (U/mg protein)	3.67 (1.61-5.76)	n = 73	4.81 (2.08-7.21)	n = 20	0.339

e-TBARS (nmol/g Hb)	29.2 (26.0-33.9)	n = 74	26.6 (22.4-29.9)	n = 26	**0.042**

e-SOD (U/gHb)	646.1 (406.9-1069)	n = 74	532.3 (437.9-970.0)	n = 25	0.429

e-CAT (U/gHb)	5122 (4510-5952)	n = 75	4749 (4235-5705)	n = 26	0.432

AOA (%)	40.6 (30.2-50.3)	n = 66	44.6 (37.4-52.3)	n = 23	0.267

FRAP (μmol/L)	775.9 (692.5-951.2)	n = 73	737.1 (625.0-839.8)	n = 26	0.112

TEAC (Trolox equivalents)	4.68 (4.20-5.10)	n = 74	4.70 (4.11-5.26)	n = 26	0.867

Data are expressed as median (interquartile range) values, and were analysed using the Mann- Whitney U test, with the exception of age which is expressed as mean ± SD and was analysed by unpaired Student's t-test

**Figure 1:**
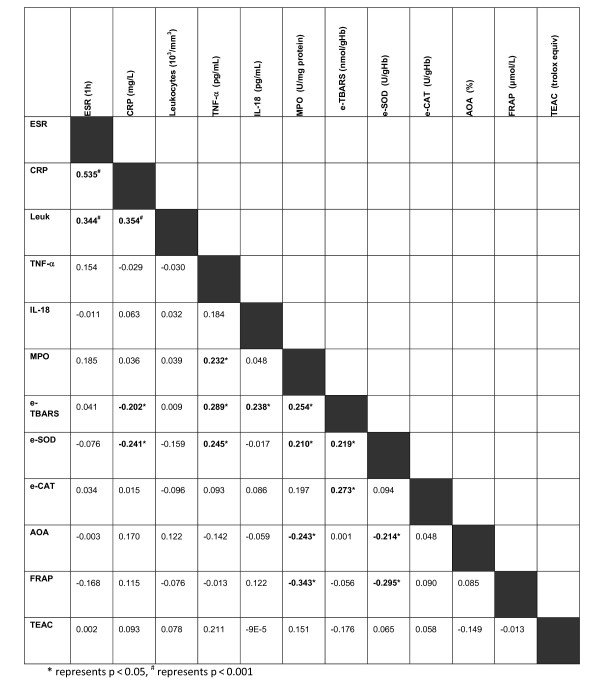
**Spearman's rank correlation coefficients (r values) for standard laboratory indices and oxidative stress-associated measures in Behcet's disease patients**.

Positive correlations were found between the concentrations of established markers of inflammation (ESR, CRP and leukocyte count) in patients with BD. R values for the correlations of all markers are given in Figure 1. Although other statistically significant correlations were observed between various parameters measured in this study, none of the correlations were as strong as those observed when correlating one inflammatory marker with another inflammatory marker, and none of the oxidative stress-associated markers were found to correlate strongly with ESR, CRP or leukocyte count. The strongest positive correlation was found between eTBARS and TNF-α (r = 0.289, p = 0.008), whilst the strongest negative correlation was observed between MPO and FRAP (r = -0.343, p = 0.001). These may indicate the close relation between oxidative stress and inflammatory processes in BD pathophysiology.

Neither inflammatory parameters nor oxidative stress-associated parameters showed any statistically significant differences when patients were evaluated in regard to ocular, vascular, articular or mucocutaneous involvement (data not shown). There were also no significant correlations between any of the measured parameters and disease duration.

## Discussion

In the present study, we explored the potential utility of measures of oxidative stress in monitoring disease activity, and in the diagnosis of complications, in BD patients. We applied a broad panel of widely adopted oxidative stress-associated measures to plasma, serum and erythrocyte samples. These measures were carried out in conjunction with both standard laboratory assays of the inflammatory response, such as CRP and ESR, and clinical assessments. We aimed to assess the additional clinical information that measures of oxidative stress might provide in terms of disease activity or the diagnosis of complications.

As expected, the group of BD patients showed evidence of a substantial inflammatory response, as indicated by high median levels of CRP, ESR, blood leukocyte count, MPO and IL-18, compared with healthy, age- and sex-matched control individuals. Moreover, when the BD group was subdivided into active disease and remission subgroups, some of these inflammatory measures were clearly elevated in the active disease subgroup compared with the remission subgroup. In contrast - and as expected - measures of inflammation provided no obvious indication of clinical complications or disease duration. Thus, our findings with standard inflammatory measures are consistent with their known nonspecific increase in BD. None of these markers can be defined to be a specific measure for disease activity or an indicator of any complication. [19]

TNF-α is an important proinflammatory cytokine and it has been implicated in the pathogenesis of many inflammatory and autoimmune diseases such as sepsis, inflammatory intestinal disease [20] and RA [21]. Similar to these studies, Oztas et al [22] have shown the role of this cytokine in BD patients. Our study found no statistically significant evidence of elevated TNF-α concentrations in serum samples from patients versus healthy subjects, nor in BD patients with active disease versus patients in remission. This might be due to the presence of other proteins released in the active period which interfere with the TNF-α measures or lack of ability of the current assay to detect TNF-α that had been complexed by the soluble TNF receptor. Our results demonstrate that serum TNF-α is not a reliable biomarker of either the presence of BD or of disease activity.

Recently, Musabak and colleagues [23] and Lee and colleagues [24] independently showed the role of another proinflammatory cytokine, IL-18, in the pathogenesis of BD. It was proposed that IL-18 probably directs the immune response in BD toward the T helper 1 type pathway. This hypothesis was supported by high serum levels of IL-18 in inactive patients, indicating an ongoing T helper polarization and subclinical inflammation during the inactive period. Interestingly, we also observed higher IL-18 levels in the BD patient group when compared with controls. In regard to IL-18 concentrations in active and remission periods, the difference between the median values in the two groups was less marked, although still statistically significant. This may be due to the subclinical inflammation during the inactive period, as proposed previously [23].

Increased neutrophil activation is observed in BD patients and this can directly be determined with MPO activities. In accordance with previous studies [25,26], leukocyte MPO activity was higher in the BD patient group than the control group in our study. In addition, the weak positive correlation found between leukocyte MPO activity and serum TNF-α in BD patients (r = 0.232, p = 0.041) demonstrates the concomitant increase in neutrophil activation and inflammation markers in BD.

The strong negative correlation between leukocyte MPO activity and FRAP (r = -0.343, p = 0.001) in this study shows the importance of the oxidant/antioxidant balance in BD patients. In inflammatory diseases such as BD, MPO production from activated neutrophils is increased, which could be consistent with oxidative stress. Also consistent with oxidative stress, plasma antioxidant activity in BD is reportedly decreased [27].

Although increased oxidative stress has been suggested to be another hallmark of BD [2,28,29], the present study provides little evidence to support this idea, despite the clearly inflamed status of the BD group. Previously, a number of studies have shown an increase in serum TBARS and eTBARS concentrations in BD patients [27,30,31]. In contrast, our study showed no evidence that eTBARS levels were higher in patients than controls.

In the present study, when patients were grouped for ocular, vascular, articular and mucocutaneous involvements, the measured parameters did not show statistically significant differences between groups. In contrast, Taysi et al. [32] have shown a high serum MDA level and SOD activity in patients with ocular involvement. We also found no correlations between measures of oxidative stress and disease duration. In this respect, our results are in concordance with other related literature [33].

It has been proposed that NO plays a role in the pathogenesis of BD. Laroux et al. [34] showed that NO modulates the adhesion molecules induced by cytokines in inflammatory processes and Gunduz et al. [35] futher suggested that increased NO release from the endothelium is expected in BD patients due to enhanced leukocyte-endothelium interactions. Previously increases in NO production due to various forms of the tissue injury in inflammation have been shown [36]. In recent years studies showed that S-nitrosothiols, formed by the reaction of reactive nitrogen species (derived from NO) with free thiols, are increased in biologic systems as a response to inflammatory reactions [5,37]. In the current study RSNOs in the plasma of BD and control subjects were not within the detection limit of the analytical technique (EPR spectrometry with spin trapping). The technique was further applied with various preanalytical and analytical modifications such as ultrafiltration, incubating at alternative temperatures and time, and enzymatic proteolysis. Despite all these modifications, RSNOs could not be detected and this is attributed to their very low levels in BD plasma. Previously, RSNOs were detected in plasma and synovial fluid from rheumatoid arthritis patients, but seldom in healthy control subjects [5].

Recently 3-NT, which is produced by nitration of tyrosine by reactive nitrogen species has been suggested to be a useful marker for inflammation and NO-mediated tissue injury [38,39]. Both the free and protein-bound nitrotyrosine forms are increased in various tissues in inflammatory disorders such as atherosclerotic plaques, intestinal tissue of inflammatory bowel disease patients, and lung tissue of adults with respiratory distress syndrome [40-42]. In our study, we aimed to determine whether the local formation of 3-NT in the inflammatory processes in BD is also systemically detectable. In our previous work investigating the 3-NT levels in RA patients [43], a proportion of both plasma and SF samples from RA patients had detectable 3-NT levels with the ELISA sandwich method used. However, in the current study no detectable 3-NT was observed in BD patients or controls with the same method. This was also in contrast to the findings of epnr et al [30].

In conclusion, in agreement with previous studies [22,23,25,27], significant increases in BD patients compared with healthy control subjects, were observed in ESR, CRP, leukocyte count, IL-18, and MPO enzyme activity, indicating the activation of leukocytes and macrophages in the inflammatory process. However, in contrast to previous studies, we found that the assays used appeared to offer no potential for providing additional insight into disease activity or the diagnosis of disease complications. RSNOs and 3-NT, which are linked to NO metabolism and are proposed to play a role in the pathogenesis of BD, were not proved to be markers for the active period of BD. We propose that the search for a biochemical marker that will indicate the active period in BD should be continued with new (more specific and sensitive) measures of oxidative stress, and in larger studies.

## Competing interests

The authors declare that they have no competing interests.

## Authors' contributions

YDA, FSG, EYS, participated in the study design and experimental work and drafting the manuscript, ET, IK and PGW, participated in the experimental work and drafting the manuscript, KA and GK provided the patients and participated in drafting of the manuscript. All authors read and approved the final manuscript.
